# Convective Flow Redistribution of Oxygen by Laser Melting of a Zr-Based Amorphous Alloy

**DOI:** 10.3390/ma16114113

**Published:** 2023-05-31

**Authors:** Inga K. Goetz, Victor Pacheco, Carl J. Hassila, Ulf Jansson, Jochen M. Schneider, Marcus Hans

**Affiliations:** 1 Department of Physics and Astronomy, Materials Physics, Uppsala University, Box 530, SE-75121 Uppsala, Sweden; 2 Materials Chemistry, RWTH Aachen University, Kopernikusstr. 10, D-52074 Aachen, Germany; schneider@mch.rwth-aachen.de; 3 Department of Chemistry-Angström Laboratory, Uppsala University, Box 523, SE-75120 Uppsala, Swedenulf.jansson@kemi.uu.se (U.J.); 4 Department of Materials Science and Engineering, Biomedical Engineering, Uppsala University, Box 35, SE-75103 Uppsala, Sweden

**Keywords:** bulk metallic glass, single laser tracks, oxygen contamination, surface oxides, atom probe tomography, transmission electron microscopy

## Abstract

Oxygen impurities play a crucial role in the glass-forming ability and crystallisation behaviour of metallic glasses. In the present work, single laser tracks were produced on Zr_59.3-x_Cu_28.8_ Al_10.4_Nb_1.5_O_x_ substrates (x = 0.3, 1.3) to study the redistribution of oxygen in the melt pool under laser melting, which provides the basis for laser powder bed fusion additive manufacturing. Since such substrates are commercially not available, they were fabricated by arc melting and splat quenching. X-ray diffraction revealed that the substrate with 0.3 at.% oxygen was X-ray amorphous, while the substrate with 1.3 at.% oxygen was partially crystalline. Hence, it is evident that the oxygen content affects the crystallisation kinetics. Subsequently, single laser tracks were produced on the surface of these substrates, and the melt pools attained from the laser processing were characterised by atom probe tomography and transmission electron microscopy. Surface oxidation and subsequent convective flow redistribution of oxygen by laser melting were identified as causes of the presence of CuO_x_ and crystalline ZrO nanoparticles in the melt pool. Bands of ZrO likely originate from surface oxides that were moved deeper into the melt pool by convective flow. The findings presented here highlight the influence of oxygen redistribution from the surface into the melt pool during laser processing.

## 1. Introduction

Metallic glasses, i.e., alloys that exhibit amorphous structures without long-range order, were first reported in the 1960s by Duwez et al. [1]. Since their discovery, these materials have attracted the interest of scientists and engineers due to their excellent mechanical and/or magnetic properties. For instance, this type of material has been used in the production of sports goods [2], jewellery [3], and transformers [4]. One of the main obstacles to the widespread use of metallic glasses has been the dimensional restrictions posed by the manufacturing techniques suitable for these substances, e.g., casting, which is affected by limited cooling rates. This translates into maximum casting thicknesses of only a few millimeters for good glass-forming systems, while larger parts cannot be produced without crystallisation taking place. In recent years, the development of layer-by-layer additive manufacturing techniques has enabled the production of metallic glasses with complex shapes and dimensions beyond what can be achieved with any other fabrication route. Of these methods, laser powder-bed fusion (LPBF, also referred to as selective laser melting) is the most widely used, and it has been employed to produce Fe- [5,6], Ti- [7] and Zr-based [8,9,10,11,12] metallic-glass-forming alloys. In particular, Zr-based metallic glasses have drawn increased attention due to their combination of excellent glass-forming abilities (GFA) and remarkable mechanical properties [13].

For Zr-based alloys, the presence of oxygen during the different stages of the LPBF fabrication remains a challenge due to the incorporation of impurities. Both the powder form of the raw material and the atmosphere in the chamber during the LPBF process contribute to high impurity levels. For Zr_59.3_Cu_28.8_Al_10.4_Nb_1.5_ (AMLOY-ZR01), an oxygen impurity of around 1 at.% was found for printed samples and could be attributed to the supplied powder, not the printing process, as no change in the oxygen concentration was observed after processing [10]. Oxygen has been reported to decrease both the GFA and the thermal stability of Zr-based alloys by facilitating the formation of crystalline particles [9,14,15,16]. In addition, Bordeenithikasem et al. found that LPBF samples with higher oxygen concentrations showed embrittlement compared to cast samples with lower oxygen levels, and the embrittlement was attributed to both porosity and the oxygen concentration [14]. Best et al. [8] reported a significant decrease in ductility and toughness due to dissolved oxygen in the structure of Zr_59.3_Cu_28.8_Al_10.4_Nb_1.5_ metallic glasses. While LPBF-processed material contained 1271 ± 41 ppm oxygen and exhibited a fracture toughness of 24–29 MPa$\sqrt{m}$, the oxygen content in the cast material was significantly lower, with 168 ± 61 ppm, and resulted in an order of magnitude higher fracture toughness [8,11]. The detrimental effect of oxygen on the fracture toughness was explained by the effect of dissolved oxygen on the glass structure, rendering shear transitions less energetically favourable [8,11]. For ZrCu/O amorphous thin films, residual stress has been found to increase upon heating due to devitrification [17]. Hence, oxygen impurities have a detrimental effect on the mechanical properties and performance of Zr-based alloys.

These and other investigations have shown the important role of oxygen in determining the final characteristics and properties of Zr-based metallic glasses produced by LPBF. Oxygen-related impurities can originate from a range of sub-processes, such as the raw materials themselves, powder production, powder storage, processing, and surface oxidation; however, the individual contribution of each factor is often not determinable. Furthermore, the exact mechanism by which oxygen is incorporated into the material from the surface of the powder particles and/or from the gas impurities in the process chamber is not fully understood.

In this work, the oxygen incorporation and redistribution in metallic glasses is investigated using single tracks on amorphous substrates. As Zr-based amorphous alloys are well studied and have been successfully fabricated by additive manufacturing [8,9,10,11,12,18], substrates with chemical compositions based on Zr_59.3_Cu_28.8_Al_10.4_Nb_1.5_ were produced by arc melting and splat quenching with two different oxygen contents (0.3 and 1.3 at.%). The attainable cooling rates in the splat-quenching process (10^5^–10^8^ K/s [19]) are comparable to those in LPBF (10^3^–10^8^ K/s [20]). The findings presented here highlight the influence of oxygen redistribution from the surface into the melt pool during laser processing, which provides the basis for the LPBF process.

## 2. Materials and Methods

### 2.1. Substrate and Sample Fabrication

**Figure 1:**
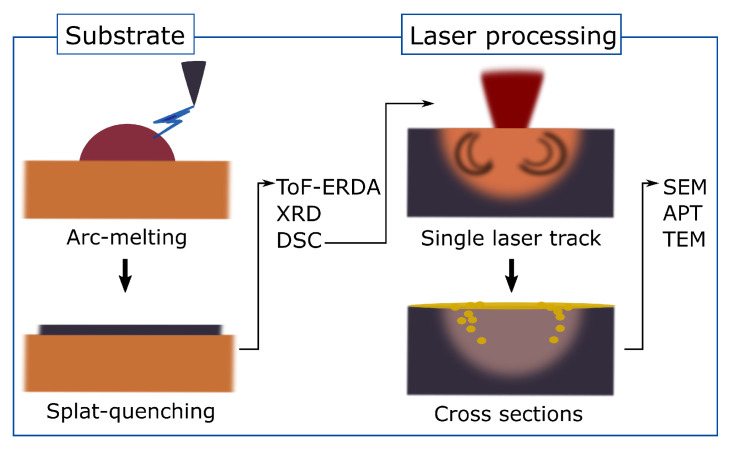
Schematic overview of processing steps and characterisation methods. Substrate fabrication by arc melting, followed by splat quenching and characterisation with time-of-flight elastic recoil detection analysis (ToF-ERDA), X-ray diffraction (XRD), and differential scanning calorimetry (DSC). Single-laser-track processing of substrate materials and analysis of cross-sections was carried out by scanning electron microscopy (SEM), atom probe tomography (APT), and transmission electron microscopy (TEM). The laser-processed substrates are referred to as samples; the term untreated sample specifies the regions away from the laser tracks, while the term laser-processed sample refers to the region melted by the laser scan.

To study the influence of oxygen content on metallic glasses in single-laser-track experiments, substrates of the amorphous alloys were needed. The chemical composition of these substrates was based on the commercially used Zr_59.3_Cu_28.8_Al_10.4_Nb_1.5_ (AMLOY-ZR01, Heraeus Holding GmbH, Hanau, Germany) [21]. As such substrates are not commercially available, they were fabricated by the arc melting of the desired composition and subsequent splat quenching to obtain an amorphous substrate. Laser tracks were then written on the surface of these substrate sheets. The process steps and characterisation techniques are summarised in Figure 1. From here on, the splat-quenched sheets prior to and after laser processing will be distinguished as the *substrates* and *samples*, respectively. The term *untreated sample* will be used to specify the regions away from the laser tracks, while the term *laser-processed sample* will be used to refer to the region melted by the laser scanning.

Two alloys with different controlled oxygen contents and nominal compositions of Zr_59.3_Cu_28.8_Al_10.4_Nb_1.5_ and Zr_58.3_Cu_28.8_Al_10.4_Nb_1.5_O_1_ were weighed in from high-purity raw elements (Thermo Fisher (Kandel) GmbH, Kandel, Germany), as follows: Zr crystal bar (oxygen < 50 ppm, 99.5% (metal basis)); Cu slugs (oxygen-free, 99.997%); Al (99.99% (metal basis)); and Nb slugs (61 ppm oxygen, 99.992%). These alloys were subsequently arc-melted. Oxygen was introduced to the composition by adding CuO in powdered form (ABSCO Material, Haverhill, United Kingdom, 99.9%). To avoid loss of the powder during arc melting, the CuO was wrapped in Cu foil (99.9999% (metal basis)). Arc melting of the alloys was performed in an Ar atmosphere after melting a Ti-getter multiple times to purify the atmosphere. The prepared compositions were subsequently arc melted again according to the same procedure and splat quenched by releasing a piston on a water-cooled copper plate. The produced substrate sheets were kept in standard sample boxes in air until the laser treatment was performed so that surface oxidation could occur.

The single-scan tracks were produced using a laser power of 75 W, which yielded additively manufactured X-ray amorphous bulk samples in a previous study on the same material in powder form [10]. With lower powers, amorphous but more porous parts were attained, while with higher powers, the porosity decreased, but the crystalline fraction increased for powers exceeding 75 W [10]. The laser writing was performed in an Ar atmosphere (purity > 99.9997%) using an EOS M100 (EOS GmbH, Munich, Germany) equipped with a Yb-fiber laser with a minimum beam diameter of 40 µm. The laser scanning was controlled by an optical scanner with a constant speed of 2000 mm/s at the focal plane. The single tracks were spaced approximately 2 mm apart to avoid any influence from adjacent tracks.

### 2.2. Analysis of Splat-Quenched Substrates

The composition of the splat-quenched substrates for the laser tracks was determined by time-of-flight elastic recoil detection analysis (ToF-ERDA) with a 44 MeV ^127^I^10+^ primary beam at the Tandem Laboratory [22]. The incoming and recoil angles were set to 22.5° and 45°, respectively. The employed chamber and detection procedure is described in [23]. Data analysis was carried out using Potku version 2.0 (University of Jyväskylä, Finnland) [24] with C, Si, TiN and AlO as reference samples. The Zr and Nb signals were not distinguishable; therefore, they are shown in sum in the results. For the nm scaling, a fixed conversion factor based on the reported density of 6.64 g/cm^3^ [10] was employed: 1000 standard ion beam units ($10^{18}$ atoms/cm^2^) correspond to 191 nm depth.

Differential scanning calorimetry (DSC) was carried out using a Netzsch DSC 204 F1 instrument (Erich NETZSCH GmbH & Co. Holding KG, Selb, Germany). To avoid undesired oxygen contamination and oxidation, the aluminium crucibles were sealed in an Ar-filled glove box before the measurements. The instrument specification states a temperature precision of ±0.01 °C and a temperature accuracy of ±0.1 °C after calibration. The measurements were conducted, employing the same calibration file and with a heating rate of 20 K/min from 20 to 588 °C. For X-ray diffraction (XRD), a D8 AdvanceTwinTwin (Bruker Corporation, Billerica, MA, USA), equipped with Cu Kα radiation and a Lynxeye XE PSD detector, was operated in Bragg–Brentano geometry.

### 2.3. Analysis of Laser-Processed Samples in Cross-Section

Cross-sections of the produced laser single tracks were achieved by mounting the exposed sheets using a spring clamp and thermosetting bakelite resin. Subsequently, the cross-section surfaces were prepared by grinding and polishing using diamond suspensions. A Zeiss Merlin (Carl Zeiss AG, Oberkochen, Germany) field-emission gun scanning electron microscope (FEG SEM) equipped with a backscatter electron BSE detector was used to reveal any inhomogeneities of the distribution of the constituent elements from the alloy within the melt pool. Areas within the melt pool appear brighter because of a relatively higher concentration of heavier elements, while lighter elements are visualised by darker areas of the micrograph.

Atom probe specimens were prepared by focused ion beam (FIB) techniques using a Helios Nanolab 660 dual-beam microscope (FEI, Hillsboro, OR, USA). Site-specific lift-outs within the region affected by the laser track as well as from the splat-quenched material (approx. 0.5 mm apart from the laser track) were carried out for untreated and laser-processed samples with 0.3 and 1.3 at.% oxygen. A standard specimen preparation protocol was employed [25], and the final cleaning step was carried out with 5 kV and 40 pA for 4 min in order to achieve a radius at the apex of <50 nm and a shank angle of approximately 20°. The prepared specimens were exposed to atmosphere for approximately 3 min due to the transport from the dual-beam to the atom probe microscope. Chemical composition characterisation was carried out at the nanometre scale by laser-assisted atom probe tomography (APT) using a LEAP 4000X HR (Cameca, Madison, WI, USA). The laser pulse energy was 60 pJ, the laser pulse frequency was 125 kHz, and the base temperature was 60 K, while the detection rate was set at 0.5 at.%. A total of 15 million ions were acquired for each of the four measurements, and the IVAS 3.8.0 software (Cameca, Madison, WI, USA) was used for data analysis, employing the shank angle reconstruction protocol. In addition, atom probe specimens were prepared from high-purity raw Zr (see Section 2.1) to examine the effect of atmosphere exposure during the transport to the atom probe microscope. Field evaporation was carried out at a laser pulse energy, frequency, and base temperature of 10 pJ, 125 kHz, and 30 K, respectively, and 2 million ions were collected.

Transmission electron microscopy (TEM) with energy dispersive X-ray spectroscopy (Titan Themis with SuperX EDS, FEI, Hillsboro, OR, USA) was employed to analyse a lamella prepared by FIB milling from the cross-section of the laser-processed sample with 0.3 at.% oxygen. In addition to the chemical information, structural information was gathered with selected area electron diffraction (SAED) and convergent beam electron diffraction (CBED).

## 3. Results

**Figure 2:**
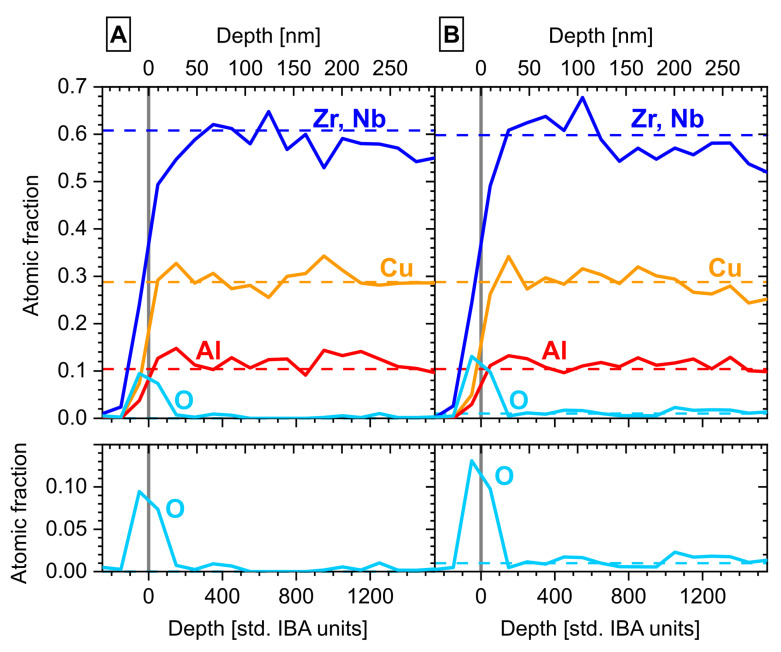
ERDA depth profiles of both substrates. (**A**) No added oxide, 0.3 at.% oxygen (excluding surface). (**B**) CuO added to composition, 1.3 at.% oxygen (excluding surface). Nominal compositions are marked with dashed lines.

ToF-ERDA measurements were performed to accurately quantify the actual compositions of both substrates. The measured depth profiles, shown in Figure 2, confirm that the bulk compositions were (Zr+Nb)_58.3_Cu_29.4_Al_11.9_O_0.3_ and (Zr+Nb)_58.6_Cu_28.8_Al_11.4_O_1.3_, which were close to the desired nominal compositions (indicated by the dashed lines in Figure 2). The oxygen level of both substrates was about 0.3 at.% higher (excluding the surface) than the nominal compositions, thus likely reflecting the impurity contribution due to arc melting and splat quenching, in addition to the impurity concentration of the raw materials. The surface shows a clear oxygen-rich layer with the signal peak centered around zero depth. The statistical errors of the ToF-ERDA results for (Zr+Nb), Cu, and Al were ±0.7 at.%, 0.5 at.%, and 0.4 at.%, respectively, indicating that the remaining discrepancies around 1 at.% likely originate from the manual weighing in of the individual materials. For oxygen, the statistical error was ±0.1 at.%. The depth profile in nm was calculated using the reported density of 6.64 g/cm^3^ for the cast material [10].

**Figure 3:**
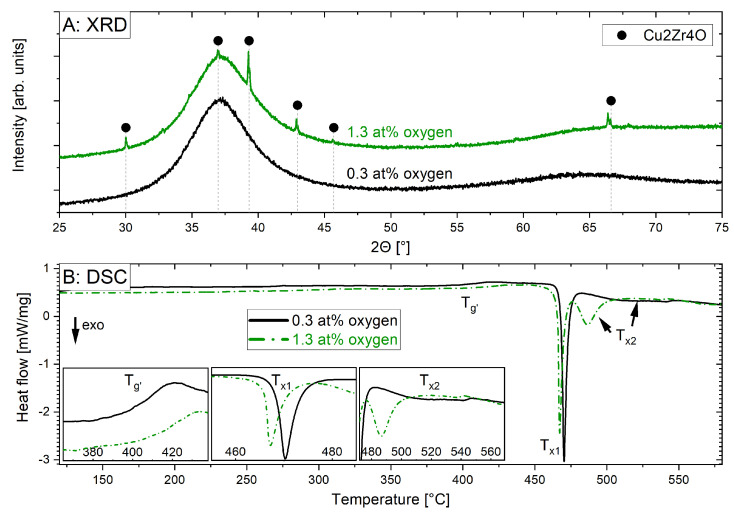
XRD (**A**) and DSC (**B**) measurements on the two substrates with different oxygen contents (0.3 at.%, 1.3 at.%). The indexed peaks are marked with the positions of Cu_2_Zr_4_O (227) with a lattice parameter of around 11.9 Å.

The structure and thermal stability of the substrates were analysed by XRD and DSC, respectively, as presented in Figure 3. The substrate with 0.3 at.% oxygen was X-ray amorphous, while the substrate with 1.3 at.% oxygen showed a small crystalline fraction. This indicates that the cooling rate of the splat-quenching process was no longer sufficient to avoid crystallisation when oxygen was added intentionally, which highlights the crucial role of comparatively small oxygen concentrations. The observed peaks in the XRD pattern can be attributed to Cu_2_Zr_4_O (space group 227), with a lattice parameter of approximately 11.9 Å, which has been observed for additively manufactured samples with similar compositions and oxygen impurity levels [9,10]. The partial crystallisation is in good agreement with the work of Kim et al., which indicated that the addition of oxygen in Zr-based metallic glasses causes a decrease in glass-forming ability [26].

The DSC scans in Figure 3 additionally indicate that the kinetics of the glass-crystalline phase transformation is faster in the substrate with 1.3 at.% oxygen. For instance, a crystallisation temperature of 466 °C was determined for this substrate, while a value of 468 °C was obtained for the substrate with 0.3 at.% oxygen. This 2 °C reduction in crystallisation temperature due to a difference in oxygen content of 1 at.% and the associated crystalline phase fraction is consistent with measurements obtained for cast and additively manufactured samples. For an additively manufactured sample, a crystallisation temperature of 471.4 °C was observed, while 472.2 °C was measured for a suction-cast sample [9]. The shift was attributed to the different processing routes and to the purity of the raw materials, as the additively manufactured samples were found to contain a higher oxygen content and Cu_2_Zr_4_O nanocrystals [9]. Although the crystallisation temperature is affected by the oxygen concentration, other factors, such as different processing strategies, also affect crystallisation. Furthermore, a secondary transformation peak (marked as T_x2_ in Figure 3) can be seen for the substrate with 1.3 at.% oxygen, which is more spread out and takes place at higher temperatures compared to the substrate with 0.3 at.% oxygen. It is relevant to mention that a plateau in the supercooled liquid region (SCLR) is only observed for the substrate with 0.3 at.% oxygen. A plateau in the SCLR has been previously associated with the capacity to effectively thermoplastically form the material [27]. The lack of a plateau in the SCLR of the substrate with 1.3 at.% oxygen could be associated with the presence of crystalline particles, as has been indicated in previous investigations [10].

**Figure 4:**
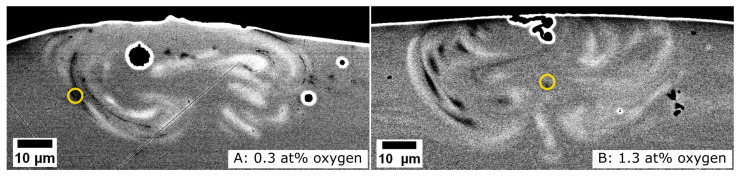
SEM backscatter images of cross-sections of laser tracks written on splat-quenched substrates. (**A**) Laser-processed sample with 0.3 at.% oxygen. (**B**) Laser-processed sample with 1.3 at.% oxygen. Approximate positions of APT lift-outs are marked with yellow circles.

The above described alloy sheets were subsequently used as substrates for single-track laser scans. The cross-sections of these laser-processed samples are shown in Figure 4. Through the use of imaging using backscattered electrons, the track geometry is clearly visible, while there is no apparent trace of the tracks when imaging with secondary electrons. The size and geometry of the visible melt pools are very similar to those observed for similar experiments on additively manufactured and cast substrates [28]. The extent of the melt pool is semicircular, without indications of keyhole formation, and only ∼33% larger than the laser beam diameter at the focus (40 µm). The black areas surrounded by intense white halos are not features of the track. The image of the sample with 0.3 at.% oxygen (A) exhibits three circular shapes that probably originate from contamination on the polished surface, while the shape in the image of the laser-processed sample with 1.3 at.% oxygen (B) is a crack on the surface of the track, into which the moulding compound flowed during embedding. The melt pool geometry exhibits a distinct flow pattern. The chemical contrast image is consistent with convective flow within the melt pool during laser processing. The light contrast appears very similar to the observations made by Lindwall et al. [28]. The chemical contrast observed for the cross-section of the laser-processed samples (Figure 4) was further studied by APT and TEM.

**Figure 5:**
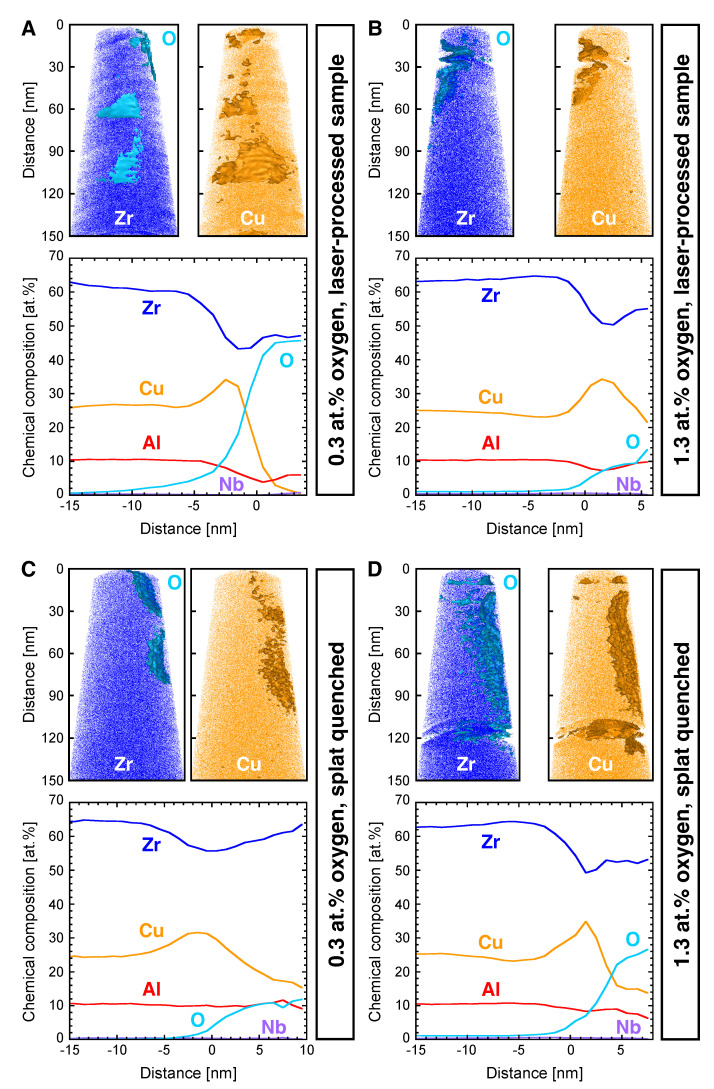
Local chemical composition measured by APT. Zr atomic positions with oxygen isosurfaces, Cu atomic positions with isosurfaces, and respective proxigrams from (**A**) laser-processed sample with 0.3 at.% O, (**B**) laser-processed sample with 1.3 at.% O, (**C**) untreated sample with 0.3 at.% O ∼500 µm from laser track, and (**D**) untreated sample with 1.3 at.% O ∼500 µm from laser track.

The local chemical composition at the nanometre scale was studied by APT for laser-processed and untreated samples with 0.3 and 1.3 at.% oxygen. The atomic positions of Zr (together with O isoconcentration surfaces) and Cu (together with Cu isoconcentration surfaces) as well as oxygen proximity histograms are shown in Figure 5. Specimens were prepared from the melt pools of the laser tracks of the sample with 0.3 at.% oxygen (Figure 5A; lift-out region indicated in Figure 4A), as well as with 1.3 at.% oxygen (Figure 5B; lift-out region indicated in Figure 4B). Moreover, the untreated samples were characterised by the preparation of tips from the splat-quenched base material at a distance of 0.5 mm from the laser tracks (Figure 5C: 0.3 at.% oxygen; Figure 5D: 1.3 at.% oxygen). Oxygen was mainly detected as molecular ZrO ions for all of the measured APT specimens. Oxygen-rich regions were observed in the upper part of all specimens (closer to the surface of the cross-section). In contrast, the oxygen content of the high-purity raw Zr, measured as a comparison, was homogeneously distributed. Thus, the exposure of the specimens to the atmosphere during transport from the dual-beam to the atom probe microscope is unlikely to cause the formation of oxygen-rich regions.

In Figure 5, oxygen isoconcentration surfaces highlight the elemental distribution around oxygen-rich nanoparticles. Here, Zr is depleted at the interface compared to the nanoparticles and matrix, while Cu is enriched at the interface. The observed different oxygen levels from the nanoparticles do not necessary reflect a chemical difference but could result from the geometrical fraction of the individual nanoparticles incorporated into the tip. Since oxygen-rich regions can be observed in the specimens from the melt pools (laser-processed samples) as well as 0.5 mm away from the laser tracks (untreated samples), it can be concluded that the incorporation of these impurities takes place irrespective of the laser processing. From the data presented in Figure 3, it is evident that the fraction of these locally confined surface oxides is too small to be detected in XRD. The laser writing, and thus the remelting of the material, can, however, lead to a redistribution of the oxygen from the oxide nanoparticles via transport of the nanoparticles themselves or their melting and an incorporation of oxygen into the matrix. These possible redistribution mechanisms were further investigated by TEM analysis on a lamella prepared from the cross-section of the melt pool of the laser-processed sample with 0.3 at.% oxygen since this substrate was identified as X-ray amorphous before laser processing (Figure 3A).

**Figure 6:**
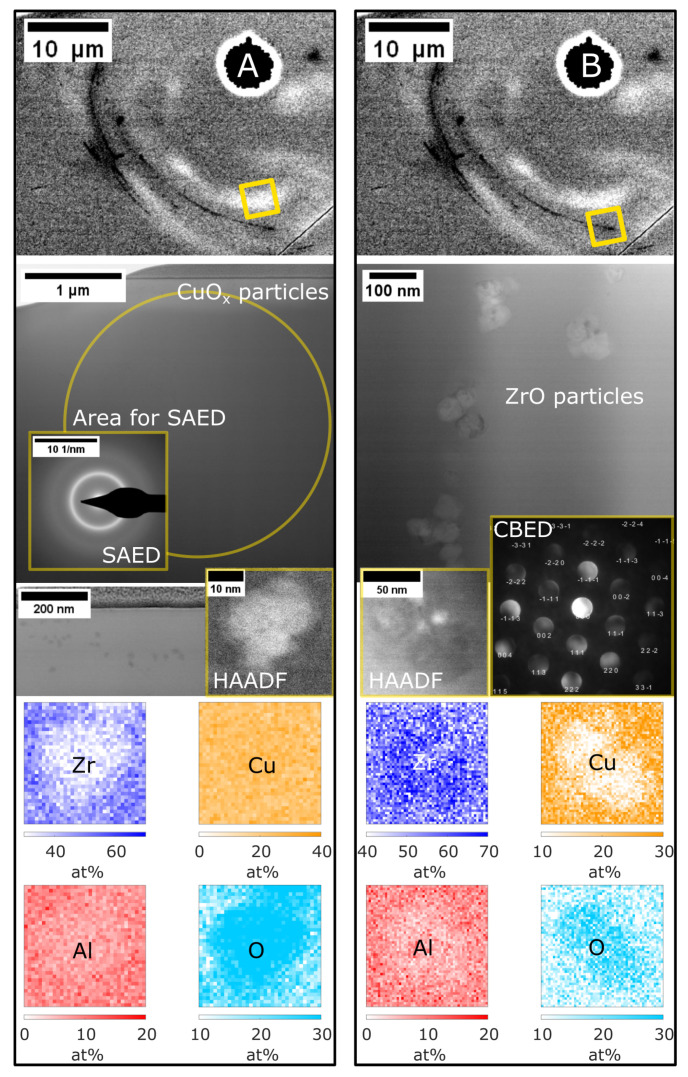
TEM analysis of the laser-processed sample with 0.3 at.% oxygen. Two areas from the obtained lamella are shown. (**A**) Bright-field STEM image and SAED show the amorphous matrix and CuO_x_ nanoparticles close to the surface of the cross-section (bright-field STEM image, HAADF STEM image of single nanoparticle and EDS). (**B**) The dark contrast from the cross-section corresponds to a band of ZrO nanoparticles (bright-field STEM image, HAADF STEM image of single nanoparticle), identified by EDS and CBED. Nb excluded due to representing a small fraction.

The two regions investigated on this lamella are depicted in Figure 6. An area with light contrast in the cross-sectional images (region A) was used to probe the matrix after laser writing. The SAED pattern, consisting of broad rings, confirms the matrix as amorphous. Toward the top of the lamella, corresponding to the surface of the cross-section of the laser-processed sample, there are nanoparticles of ∼20 nm diameter visible. EDS showed that these nanoparticles were enriched in copper and oxygen, indicating the formation of CuO_x_. While phase identification was not possible due to the small volume fraction of CuO_x_ nanoparticles, the contrast differences in the HAADF image (Figure 6A) indicate the formation of cubic CuO (space group 225, rock salt prototype). The bright appearance of the nanoparticle compared to the amorphous matrix corresponds to mass contrast and demonstrates that the CuO_x_ nanoparticles have a higher density than the matrix. Among the known Cu-O phases, cubic CuO is the only one that exhibits a higher density (6.91 to 7.36 g cm${}^{-3}$ [29]) than the presently studied sample (6.64 g cm${}^{-3}$ [10]). The second area analysed (region B) corresponds to a dark contrast band observed by imaging backscattered electrons, which indicates that the region exhibits a higher concentration of lighter elements. This dark contrast was verified to be caused by bands of nanoparticles of ∼50 nm size, as visible in the bright-field image. EDS measurements on these nanoparticles disclose the composition as Zr- and O-rich. CBED confirmed ZrO (space group 225, rock salt prototype) as a good fit for the diffraction pattern. α-Zr, which has been observed as oxygen-rich nanocrystals by [10], as well as ZrO_2_ phases were a poor fit for the CBED pattern, strengthening the finding that the chemical contrast is caused by ZrO instead. The formation of ZrO has been observed within flash-sintered yttria-stabilised zirconia as a secondary phase, and based on the reported experimental lattice parameter, a density of 6.90 g cm${}^{-3}$ can be derived [30]. However, the slightly darker appearance of the ZrO nanoparticles (Figure 6B) compared to the matrix suggests a lower mass density than the presently studied sample (6.64 g cm${}^{-3}$ [10]) and may be explained by the absence of yttrium and the presence of contributions of copper as well as aluminium, which could reduce the density of the ZrO nanoparticles.

## 4. Discussion

The different oxygen contents of the two substrate materials clearly influenced the glass formation, as seen in Figure 3. While the cooling rate of the splat-quenching process was sufficiently high to obtain an amorphous material with 0.3 at.% oxygen, as shown by X-ray diffraction and SAED, the formation of a crystalline-phase fraction was observed in the substrate with 1.3 at.% oxygen. Oxygen has been reported to decrease the GFA of Zr-based metallic glasses in previous studies [9,14,15,16]. The single laser tracks on both substrates did nevertheless result in comparable convective flow patterns (Figure 4) and, in both cases, oxygen-rich nanoparticles were identified by APT (Figure 5). The loss in GFA due to the addition of oxygen (Figure 3) can be circumvented by the high cooling rates in the LPBF process, for which industrial grade powder with a similar oxygen concentration as in the substrate with 1.3 at.% investigated here was successfully used to fabricate X-ray amorphous parts [10,14]. This is consistent with the results of Jonas et al. [31], who found that there are no significant changes in viscosity and density between the industrial and high-purity modification of the alloy but found a reduction in the undercooling ability for lower purities.

CuO_x_ nanoparticles and crystalline ZrO nanoparticles were identified by TEM (Figure 6), where the latter were observed as dark contrast bands in the cross-sections of the melt pools (Figure 4). These nanoparticles have not been observed in melt pools of similar tracks written on additively manufactured substrates [28]. The substrates in ref. [28] were, however, polished prior to the laser treatment, whereas the substrates in the present study were not. Additionally, the different fabrication routes might have led to a different degree of surface oxides, even if both the additive manufacturing and the splat-quenching techniques employed here were carried out in an Ar atmosphere. It is, therefore, plausible that these crystalline nanoparticles arise from surface oxides and are then moved into the melt pool by convective flow, while the corresponding oxides were most likely removed before processing by polishing in the study by Lindwall et al. [28]. As the usual processing condition in LPBF, for which laser melting as investigated here provides the basis, is the laser melting of powder particles with a high surface area and therefore considerable amounts of surface oxides, the present case can provide additional insights into the processing of the powder.

With the observed oxide nanoparticles present in the melt pool, an influence on the properties of an additively manufactured build has to be expected. The oxides may act as nucleation sites for other crystalline phases during further processing; this may occur, for example, by the melting of subsequent layers. Nucleation sites, for example, short-range order structural heterogeneities, have been proposed as a crucial factor for the devitrification of metallic glasses [32,33]. For Zr-based metallic glasses, the formation of oxygen-impurity-induced metastable crystalline phases has been found to act as heterogeneous nucleation sites [34], which leads to a decrease in thermal stability [9]. The oxide nanoparticles observed in the present case may thus substantially change the nucleation and growth of crystalline phases in the amorphous matrix. Based on the present observations, this contribution can be systematically evaluated by a combination of APT and TEM on materials of different oxygen contents and oxide nanoparticle fractions in further studies.

Other possibilities for the origin of the crystalline nanoparticles are a reactive formation during processing due to remaining oxygen impurities in the atmosphere or their formation within the melt. While all three origins (surface oxides, reactive formation on the surface, and formation with the available oxygen in the melt pool) could take place simultaneously, oxygen incorporation during the transportation of the atom probe specimens from the dual-beam to the atom probe microscope can be excluded. As a striking difference to the tracks presented in the study on polished substrates [28], surface oxides are highlighted as an additional prominent factor in the present study, since the other two mechanisms (reactive formation on the surface, formation with the available oxygen in the melt pool) should have taken place on a similar level in the process. Another indication is the temperature distribution within the melt pool obtained from finite element simulations of single laser tracks based on material data and characterisation of the experimentally obtained melt pool geometry [28]. At a 1 µm distance from the melt pool, a temperature of below 1000 °C is predicted for this alloy under the employed processing conditions [28]. The observed dark contrast bands appear predominantly at the outer sides of the melt pool pattern less than 10 µm from the apparent sides of the melt pool. The temperatures at these positions are thus unlikely to exceed 2000 °C [35], which would be necessary to melt the ZrO nanoparticles swept in from the surface.

Beyond the implications of the observed segregation in the melt pool for the use of the material in LPBF, the identified pattern also gives the opportunity to reflect on the melt pool dynamics and convective flow within the melt pool during processing. The nanoparticles give rise to contrast and, therefore, act as tracers. This feature could be used to study melt pool flow in comparison to simulations. With a number of cross-sectional images or tomography techniques coupled with electron backscatter imaging, the melt pool features could be studied in more detail. Possible smaller features that set the present compositions apart could be investigated in this way as well, taking into consideration the possibility that these are not distinguishable from the variations within the same track.

## 5. Conclusions

Two substrates of Zr_59.3−x_Cu_28.8_Al_10.4_Nb_1.5_O_x_ with oxygen contents of x = 0.3 at.% and x = 1.3 at.% were prepared by arc melting and splat quenching. Here, 0.3 at.% oxygen originates from impurities of the raw materials and processing. For 1.3 at.% oxygen, CuO was added in powder form to the composition. Chemical, structural, and thermal analysis of these substrates revealed that the substrate with 0.3 at.% oxygen was X-ray amorphous, while the substrate with 1.3 at.% oxygen was partially crystalline. Hence, it is evident that the crystallisation kinetics are correlated with the oxygen content.

These substrates were used to study the effect of single laser tracks prepared in a commercial laser powder bed fusion system in cross-section. The tracks on both laser-processed samples showed very similar melt pool shapes. Furthermore, the surface oxidation was identified as a prominent factor in addition to the oxygen content of the base material. Atom probe tomography revealed that both the cross-sections of the laser-processed samples and the cross-sections of the untreated samples contained zirconium and oxygen-enriched nanoparticles. These results suggest that oxide particles from the surface are moved into the melt pool during laser processing. The particles within the melt pool were investigated with transmission electron microscopy for the laser-processed sample with 0.3 at.% oxygen, revealing bands of crystalline ZrO nanoparticles. These bands likely originated from the surface and were moved into the melt pool by convective flow.

These results highlight the influence of surface oxidation on powder-based additive manufacturing processes. With similar experiments on a powder bed, the consequences for processing from powder can be analysed further in the future, including, for example, the effect of the oxide nanoparticles as heterogeneous nucleation sites on the thermal stability. Furthermore, analysing the redistribution of surface oxides provides the possibility to study melt pool dynamics. In combination with nanoindentation mapping of the melt pool cross-sections, the impact of melt pool dynamics on the mechanical properties could be explored in future studies.

## Data Availability

The data supporting the findings of this study are available from the corresponding author upon reasonable request.
